# Development of the First Patient‐Reported Experience Measure (PREM) for Hearing Loss in Audiology Care—My Hearing PREM

**DOI:** 10.1111/hex.70510

**Published:** 2026-04-01

**Authors:** Kris English

**Affiliations:** ^1^ University of Akron Akron Ohio USA

I write to express concern about two issues associated with Smith et al.'s [1] article, “Development of the first Patient‐Reported Experience Measure (PREM) for hearing loss in audiology care—My Hearing PREM.” *Health Expectations*, *27*(6), e70088.

**Issue #1:** As an audiologist, this title caught my eye, but I quickly realised that the understanding of a PREM in this article was inconsistent with conventional usage. From the abstract onward, the term ‘experience’ here applies to ‘the challenges experienced when living with a chronic condition’ (p. 1). However, a patient's ‘lived experience’ is not why PREMs have been created. From their inception, per Gilmore et al.'s [2] systematic review, PREMs are designed to give patients the opportunity to report on their clinical encounter experiences, i.e., to ‘record patient perceptions about various elements of the healthcare they received’ (p. 1). Elements of healthcare within a clinical encounter might include ease of making appointments, being treated with courtesy and respect, being heard, receiving easy‐to‐understand answers to questions, etc. Data collected from PREMs are used as indicators of healthcare quality from the patient's perspective (Bull, 2019).

An earlier systematic review about PREMs, which is included in Smith et al.'s references, provides a comparable definition: ‘Patient‐reported experience measures (PREMs) are tools that capture “what” happened during an episode of care, and “how” it happened from the perspective of the patient… [they] can provide information regarding the patient‐centeredness of existing services’ ([3], pp. 1023–1024).

Three citations were given to support the premise that PREMs ‘capture the experience of living with a health condition and receiving care’ (p. 2); however, consistent with the definitions above, the PREMs reported in these studies almost exclusively focused on receiving care, not living with a health condition. For example, all 99 items in Graham et al.'s [4] PREM designed for emergency departments address the care experience; for example, ‘Pain relief medicine was brought to me quickly; staff were thorough and paid attention to the finer details; the A&E team were respectful and polite.’

Summary: The authors do not provide a rationale for redefining ‘patient experience’ as ‘the lived experience of hearing loss’ (p. 2) in the context of a PREM. As designed, it does not meet the purpose of a PREM, which is to provide patient feedback regarding the quality of clinical encounters. Simply put, the Smith et al.'s PREM instrument is not in accord with established definitions.

**Issue #2:** From Smith et al., p. 2: ‘Our vision for the PREM is to facilitate lifeworld‐led dialogue between patients and staff, focusing on the patient experience of hearing loss so that we can provide a mechanism through which patient‐centred and lifeworld‐led approaches can be integrated into audiology care.’

The implication is that, to date, there are no such tools to facilitate this kind of dialogue. However, tools designed for this purpose have existed at least as early as 1980, when Schow and Nerbonne [5] created the ‘Self‐Assessment of Communication (SAC)’ and the ‘Significant Other Assessment of Communication (SOAC)’ (currently found in most audiologic rehabilitation textbooks). These measures have inspired the development of many more tools throughout the decades, up to and including (until recently) the innovative tools created with relevant focus groups by the Ida Institute – intentionally designed not as scorecards but as invitations to talk about those lived experiences and to create space for audiologic counselling.

Summary: The Smith et al. PREM (focusing on the experience of living with hearing loss) may be the newest dialogue‐supporting tool, but it is inaccurate to say it is the first.

I do appreciate the time and effort invested in this project, and another tool to support ‘lived experience’ conversations is always helpful – and audiology also needs a PREM that captures ‘what happened during an episode of care, and how it happened’ to inform our goal of providing measurable person‐centred care. I welcome the opportunity to communicate with the authors about these concerns.

Kris English.

## Conflicts of Interest

The author declares no conflicts of interest.

## Data Availability

The author has nothing to report.
